# Can surface landmarks help us identify the gibson interval during surgical hip dislocation: a study of 617 hips

**DOI:** 10.1007/s00402-024-05622-w

**Published:** 2024-12-18

**Authors:** Veerle Franken, Stefan Blümel, Joseph M. Schwab, Moritz Tannast

**Affiliations:** 1 Department of Orthopedic Surgery and Traumatology, HFR Cantonal Hospital, University of Fribourg, Fribourg, Switzerland; 2 Department of Orthopaedic Surgery and Traumatology, Inselspital, Bern University Hospital, University of Bern, Bern, Switzerland

**Keywords:** Gibson approach, Hip surgery, Anatomy, CT scan, Conservative hip surgery

## Abstract

**Introduction:**

The Gibson approach, used in hip-preserving surgery, is intermuscular and develops the space anteriorly to the gluteus maximus. Reliable anatomical landmarks for the development of this interval do not exist, but the interval is marked by perforating vessels (PV) of the inferior gluteal artery. The aim of this study was to provide reference values for the relationship between palpable anatomical landmarks on the femur/pelvis and the anterior border of the gluteus maximus using CT scans of the proximal femur.

**Materials and methods:**

Single center retrospective study of 617 hips who underwent a CT-scan of the pelvis/femur. We defined 5 anatomical markers on the pelvis and proximal femur and measured the distance of the anterior border of the gluteus maximus in relation to the marker, which was either anterior or posterior. The amount PV’s and it’s location relative to the innominate tubercle were measured in the coronal plane. For each landmark we compared these subgroups: male vs female, age < 40 vs ≥ 40, categorical age (< 20; 20–40; 40–60; > 60), and categorical femoral torsion (< 10°; 10°–25°; 25°–35°; > 35°).

**Results:**

Mean location of the parameters A-E was at − 8.1 cm, 1.1 cm, 1.8 cm, 1.3 cm and 0.4 cm. Parameters B, C, and D were more posterior in the age ≥ 40 group. Parameters A–E were significantly more posterior in the age > 60 group. Parameters A and E were significantly more anterior in females than in males. 50% of the PV are found between 5 and 9 cm proximal to the innominate tubercle. No statistically significant differences were noted in the location of any of the perforating vessels in the different subgroups.

**Conclusion:**

The Gibson interval is located more anteriorly in female patients and patients under 40 years of age. It is located more posteriorly in patients over 60 years of age. In addition, the interval moves anteriorly with increasing femoral torsion, most notably in patients with very high femoral torsion (> 35°).

**Supplementary Information:**

The online version contains supplementary material available at 10.1007/s00402-024-05622-w.

## Introduction

### Background

Surgical hip dislocation is, next to arthroscopic correction, used in open hip preserving surgery. Compared to arthroscopic correction, surgical hip dislocation has advantages in patients who need acetabular correction, have circumferential pathologies and/or need torsional correction at the same time [1].

The original description off the surgical hip dislocation [10] included a curved skin incision with splitting of the proximal fibers of the gluteus maximus, much like the Kocher-Langenbeck approach. EMG analysis [3], however, showed denervation of the gluteus medius and maximus in up to 53.5% and 71.4%, whenever this intramuscular plane was used. When using a straight incision, the same space can be accessed from the anterior border of the gluteus maximus muscle as described by Gibson [10, 19]. This modification of using the interval between the medius and the maximus muscle (Gibson interval) has been used regularly for surgical hip dislocation since approximately 2005. With this modification, surgical hip dislocation follows strictly internerval and intermuscular planes. Subsequently follow-up MRIs have not shown any signs of atrophy or fatty muscle infiltration [12].

Intraoperative identification of the anterior border of the gluteus maximus can be difficult. Besides trial incisions over the maximus fascia, the most reproducible anatomical landmarks are the perforating vessels of the inferior gluteal artery [2, 8]. These vessels run in the internal fascial sheet of the gluteus maximus and perforate the fascia lata between the medius and the minimus muscles. To date, no reliable topographical landmarks have been presented to find reliable surgical landmarks.

Our questions were: what is the relationship between select topographical anatomic landmarks and the anterior border of the gluteus maximus on 3-dimenional multiplanar reconstruction computed tomography (3D-MPR CT)? Does this relationship differ when categorizing patients by age, gender, and/or femoral torsion? What is the relationship between the perforating vessels normally visible at the anterior border of the gluteus maximus, and the innominate tubercle on 3D-MPR CT? Does this relationship differ when categorizing patients by age, gender, and/or femoral torsion?

The aim of this study was to correlate anatomic topographical landmarks with the location of the Gibson interval. By establishing relationships between topographical anatomy and the anterior border of the gluteus maximus, we hope to provide information that can help surgeons avoid unnecessary neurovascular and/or soft tissue damage, guide correct incision placement, and minimize inappropriate soft tissue dissection. This, in turn, will help to maximize post-operative results in patients undergoing hip-preserving surgical treatment.

It was hypothesized that elevated femoral torsion places the femur more posteriorly in regard to the Gibson interval, decreased femoral torsion places the femur more anteriorly in regard to the Gibson interval. No difference was to be expected in the location of the Gibson interval between male and female patients, and between younger and older patients.

## Material and methods

### Study design and setting

This is a single-center, retrospective, descriptive, 3D-MPR CT-based study performed at a university-based orthopedic clinic that acts as a referral center for patients with hip disease requiring tertiary care, as well as primary trauma care and osteoarthritis of the hip.

### Ethical approval

This study was approved by our institutional review board (CER-VD 2021–02054).

### Patients

**Fig. 1:**
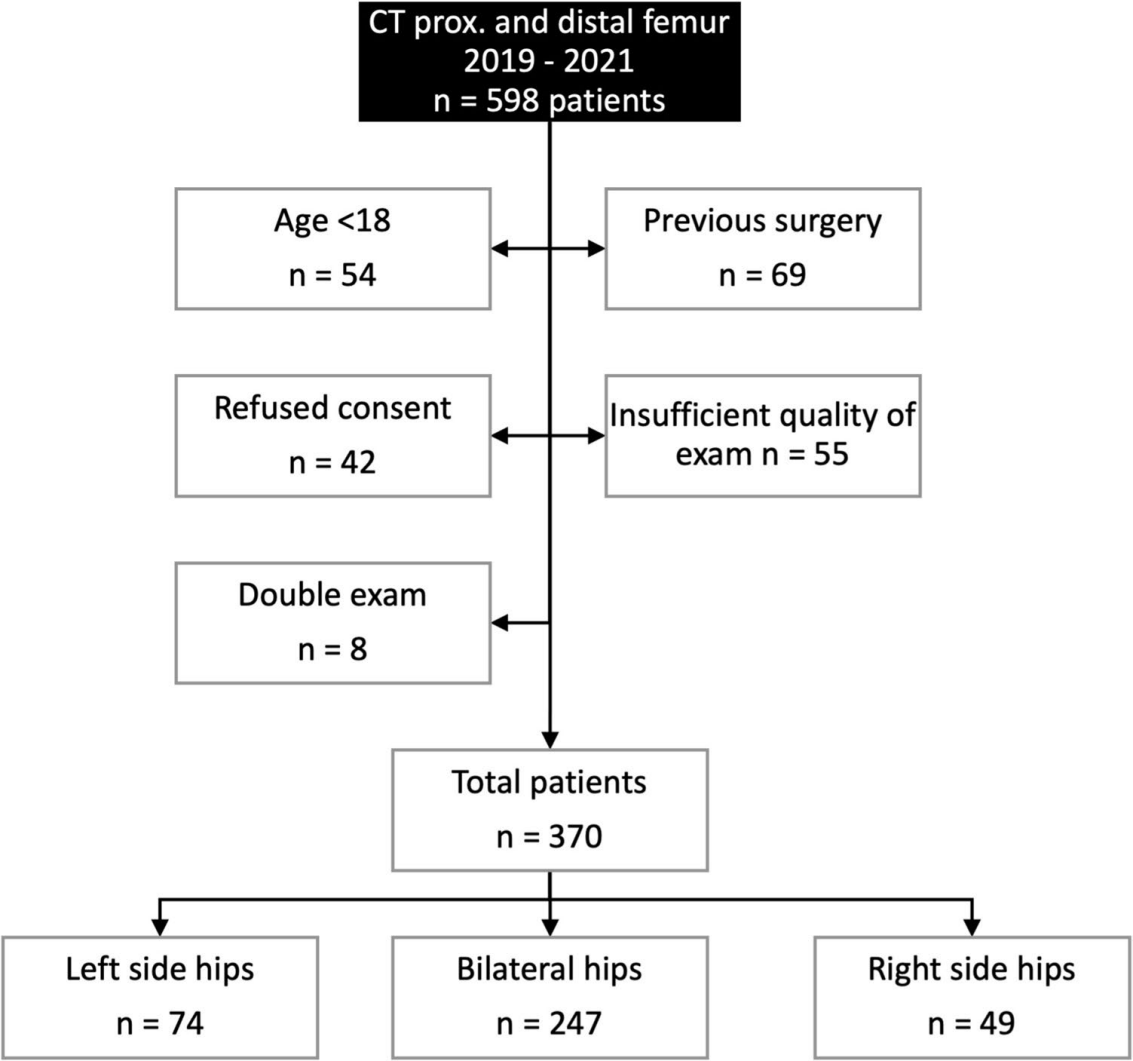
Flow chart representing inclusion and exclusion criteria

We queried our institutional picture archiving and communication system (PACS) for long axis (including pelvis, proximal femur, and distal femur) CT scans of the pelvis, hip and/or knee between 2019 and 2021 (Fig. 1). All CT scans were performed according to the same protocol. The lower limbs were positioned in 15° internal rotation to compensate for femoral torsion.

598 consecutive exams were reviewed. Exams were excluded if they were of insufficient quality (n = 55), patients were under 18 years of age (n = 54), patients refused consent (n = 42), or both hips had previously undergone surgery (n = 69). In addition, repeated CT exams of the same patient during the study period (n = 8) were excluded, and only the first exam was chosen. This resulted in 370 eligible exams to be included in our review. Of those 370 exams, both hips were eligible for inclusion in 247 (n = 494 hips). In a further 123 exams only one hip (n = 123 hips) was eligible for inclusion as the contralateral hip was excluded for having undergone previous surgery. After applying all inclusion and exclusion criteria, we were left with a total of 370 exams (370 unique patients) and 617 hips available for measurement. Osteoarthritis was not evaluated in the final patient group. Demographic and radiographic parameters were collected from our institutional electronic health record and PACS (Table 1). Mean age was 39.7 years, mean femoral torsion was 21.7º, and 51% of the hips belonged to female patients.

**Table 1:** Demographic parameters of the study group

Number of hips (patients)	617 (370)
Gender (F: M)	314 Female: 303 Male (50.9%: 49.1%)
Age (years)	39.7 ± 16.1 (18–88)
Laterality	Left (only): 74 hips Right (only): 49 hips Bilateral: 247 (494 hips)
Femoral torsion (degrees)	21.7° ± 10.5° (0º–70°)
Height (cm)	171.5 ± 9.9 (148–196)
Weight (kg)	75.1 ± 17.4 (43–140)
BMI (kg/m^2^)	25.5 ± 5.1 (16.8–44.1)

Continuous data are expressed as mean ± SD and range in parentheses

### Study variables

**Fig. 2:**
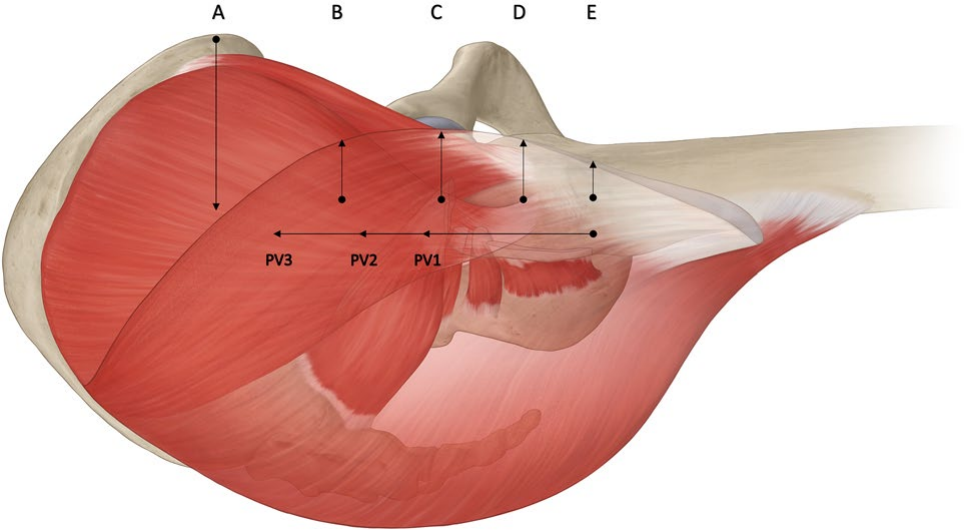
Parameters **A** ASIS, **B** ‘mid distance’ between **A** and **C**, **C** tip of trochanter, **D** center of trochanter, **E** innominate tubercle. Perforating vessel (PV) 1: first visible perforating vessel of the anterior border of gluteus maximus, measured from the innominate tubercle to proximal. Each PV is measured separately

Five anatomic reference landmarks were identified from which we could measure the radiographic distance to the anterior border of the gluteus maximus muscle (Table 2 and Fig. 2). Three of these reference landmarks, the anterior superior iliac spine (ASIS; parameter A), the tip of the greater trochanter (parameter C), and the innominate tubercle (parameter E), represent palpable topographical landmarks on a patient in the lateral decubitus position. The other reference landmarks, the “mid distance point” (parameter B) and the “trochanteric center point” (parameter D) were chosen as identifiable landmarks on CT that lie between the palpable landmarks. These additional parameters were chosen to provide a more detailed representation of the Gibson interval.

**Table 2:** Study variables

Parameter	Definition
A	Distance from anterior superior iliac spine (ASIS) to the anterior border of the gluteus maximus
B	Distance from the “mid-distance point” (defined as the midpoint between the ASIS and trochanteric tip in the coronal plane, and in plane with the trochanteric tip in the sagittal dimension) to the anterior border of the gluteus maximus
C	Distance from the trochanteric tip to the anterior border of the gluteus maximus
D	Distance from the “trochanteric center point” (defined as the midpoint of a line from the trochanteric tip to the innominate tubercle point) to the anterior border of the gluteus maximus
E	Distance from the center of the innominate tubercle to the anterior border of the gluteus maximus
Perforating vessel	High signal intensity vascular structure perforating the anterior border of the gluteus maximus and clearly traceable into subcutaneous tissue (one or more vessels may be present)
PV	Distance from the point where the vessel perforated the anterior border of the gluteus maximus and the innominate tubercle (each perforating vessel measured separately)

**Fig. 3:**
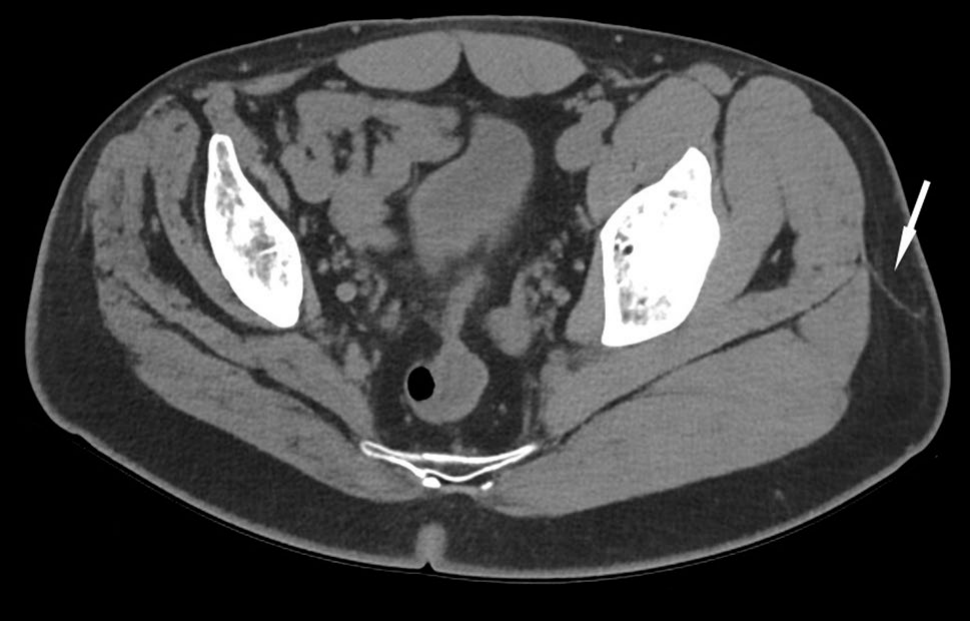
Perforating vessel (white arrow) that perforates the anterior border of the gluteus maximus, in an axial CT scan of the pelvis

**Fig. 4:**
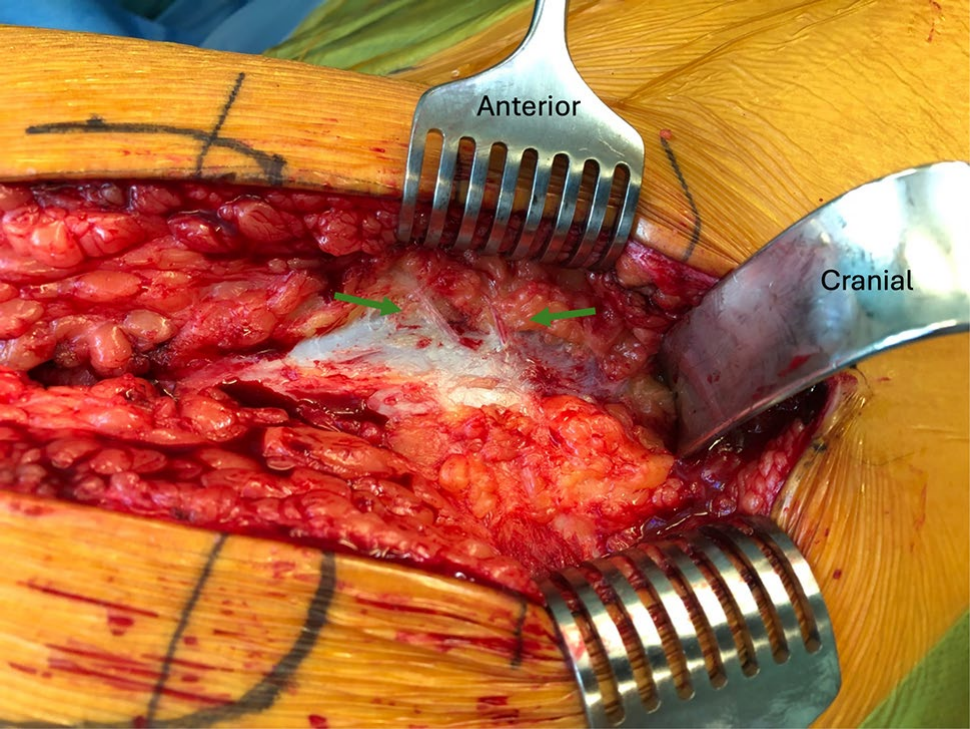
Intra-operative image of the perforating vessels (green arrows) of the inferior gluteal artery

The anatomical landmarks were identified in the sagittal, coronal, and axial planes of each 3D-MPR CT-scan (Centricity™ Universal Viewer V6.0, GE Healthcare). The distances (in cm) between each landmark and the anterior border of the gluteus maximus were measured in the axial plane. When the anterior border of the gluteus maximus was located anterior to the reference landmark, distances were recorded as positive and, likewise, those located posterior to the reference landmark were recorded as negative. In addition to our anatomic landmarks, each imaging study was evaluated to identify and locate the perforating vessel(s) (Figs. 3, 4). A perforating vessel was defined as a high signal intensity vascular structure that could be seen perforating the anterior border of the gluteus maximus and clearly continued into at least half of the subcutaneous tissue depth (Table 2). The distance (in cm) between the point where the vessel perforated the anterior border of the gluteus maximus and the innominate tubercle was measured in the coronal plane. Only vessels visible between the upper border of the lesser trochanter and the anterior superior iliac spine were included.

### Interobserver reproducibility and intraobserver reliability

All parameter and vessel measurements were performed by one observer. To assess interobserver reproducibility and intraobserver reliability, thirty scans were randomly selected to be measured a second time by the original observer, as well as by a second observer [13]. In our analysis of interobserver reproducibility, mean ICC was 0.8669 (excellent) for parameter A, 0.5344 (fair) for parameter B, 0.8492 (excellent) for parameter C, 0.5123 (fair) for parameter D, 0.8169 (excellent) for parameter E. Our analysis of intraobserver reliability showed that mean ICC was 0.8777 (excellent) for parameter A, 0.9186 (excellent) for parameter B, 0.8963 (excellent) for parameter C, 0.7007 (excellent) for parameter D, 0.8807 (excellent) for parameter E.

In our analysis of interobserver reproducibility, mean ICC was 0.5792 (fair) for parameter PV1, 0.7487 (excellent) for parameter PV2, 0.6372 (good) for parameter PV3, 0.8857 (excellent) for parameter PV4. Our analysis of intraobserver reliability showed that mean ICC was 0.8733 (excellent) for PV1, 0.9217 (excellent) for PV2, 0.9030 (excellent) for PV3, 0.8918 (excellent) for PV4.

### Subgroup analysis

In addition to analyzing our study variables for our overall group, we also generated subgroups to perform comparative analyses: male vs female, age < 40 vs age ≥ 40, categorical age (< 20; 20–40; 40–60; > 60), and categorical femoral torsion (< 10°; 10°–25°; 25°–35°; > 35°). Demographic and radiographic parameters of the subgroups are noted in Tables 3, 4, 5.

**Table 3:** Age subgroup demographics

Demographics	Age							
	Age < 40	Age ≥ 40	p-value	< 20	20–40	40–60	> 60	p-value
Number of hips (patients)	343 (183)	274 (187)		36 (19)	307 (164)	190 (115)	84 (72)	
Gender (F: M)	161 F: 182 M (46.9%: 53.1%)	153 F: 121 M (55.9%: 44.1%)		16 F: 20 M (44.4% to 55.6%)	145 F: 162 M (47.2.%: 52.8%)	106 F: 84 M (55.8%: 44.2)	47 F: 37 M (56.0%: 44.0)	
Age (years)	28.1 ± 6.1 (18–39)	54.2 ± 12.2 (40—88)	< 0.001	18.8 ± 0.4 (18 – 19)	26.2 ± 5.4 (20—39)	46.9 ± 5.5 (40—59)	70.5 ± 7.9 (60—88)	< 0.001
Laterality	Left (only): 16 hips Right (only): 7 hips Bilateral: 160 (320 hips)	Left (only): 58 hips Right (only): 42 hips Bilateral: 137 (174 hips)		Left (only): 1 hip Right (only): 1 hip Bilateral: 17 (34 hips)	Left (only): 15 hips Right (only): 6 hips Bilateral: 143 (286 hips)	Left (only): 23 hips Right (only): 17 hips Bilateral: 75 (150 hips)	Left (only): 35 hips Right (only): 25 hips Bilateral: 12 (24 hips)	
Femoral torsion (degrees)	20.2° ± 9.7° (0°–61°)	19.9° ± 9.4° (1°–48°)	0.6138	22.3° ± 8.2° (3ª–39º)	20.0° ± 9.8° (0º–61)	24.4° ± 11.3° (1º–70º)	22.0° ± 10.6° (1º–45º)	< 0.001
Height (cm)	173.1 ± 9.5 (155–193)	176.7 ± 8.5 (159–196)	0.0052	174.7 ± 7.3 (163–182)	173.0 ± 9.6 (155–193)	171.4 ± 10.4 (148–196)	167.0 ± 8.7 (149–186)	< 0.001
Weight (kg)	72.3 ± 15.3 (43–123)	87.2 ± 18.6 (18.6–140)	0.0011	66.6 ± 11.6 (51–90)	72.8 ± 15.5 (43–123)	78.2 ± 19.9 (50–140)	78.5 ± 17.5 (43–125)	< 0.001
BMI (kg/m^2^)	24 ± 4.1 (16.8–39.8)	27.7 ± 5.0 (20.4–44.1)	< 0.001	21.9 ± 3.2 (19–28.1)	24.2 ± 4.1 (16.8–39.8)	26.5 ± 5.5 (18.4–43.6)	29 ± 6.7 (21.8–44.1)	< 0.001

**Table 4:** Gender subgroup demographics

Demographics	Male	Female	p-value
Number of hips (patients)	303 (186)	314 (184)	
Age (years)	38.3 ± 15.9 (18–88)	41.0 ± 16.2 (18–88)	0.0395
Laterality	Left (only): 45 hips Right (only): 24 hips Bilateral: 117 (234 hips)	Left (only): 29 hips Right (only): 25 hips Bilateral: 130 (260 hips)	
Femoral torsion (degrees)	19.5° ± 9.2° (0º–48°)	23.9° ± 11.2° (0°–70°)	< 0.001
Height (cm)	178.3 ± 7.8 (159–196)	165 ± 6.9 (148–183)	< 0.001
Weight (kg)	83.2 ± 16.4 (51–140)	67.5 ± 14.8 (43–121)	< 0.001
BMI (kg/m^2^)	26.1 ± 4.6 (18.3–44.1)	25 ± 5.5 (16.8–43.6)	0.0338

**Table 5:** Femoral torsion subgroup demographics

Demographics	< 10°	10°–25°	25°–35°	> 35°	p-value
Number of hips (patients)	75 (62)	308 (229)	169 (131)	65 (53)	
Gender (F: M)	32 F: 43 M (42.7%: 57.3XX%)	135 F: 173 M (43.8%: 56.2%)	100 F: 69 M (59.2%: 40.8%)	44 F: 17 M (67.6%: 26.2%)	
Age (years)	38.6 ± 16.1 (19–82)	39.4 ± 16.5 (18–88)	39.1 ± 15.4 (18–88)	43.7 ± 15.6 (18–78)	0.175
Laterality	Left (only): 25 hips Right (only): 24 hips Bilateral: 13 (26 hips)	Left (only): 88 hips Right (only): 62 hips Bilateral: 79 (158 hips)	Left (only): 43 hips Right (only): 50 hips Bilateral: 38 (76 hips)	Left (only): 21 hips Right (only): 20 hips Bilateral: 12 (24hips)	
Height (cm)	172.1 ± 8.7 (155–192)	172.1 ± 12.3 (148–196)	171.1 ± 10.2 (150–196)	168.4 ± 8.3 (156–187)	0.152
Weight (kg)	80.8 ± 19.2 (53–125)	76.2 ± 17.2 (48–140)	72.4 ± 17.1 (43–130)	69.2 ± 14.2 (43–112)	0.001
BMI (kg/m^2^)	27.2 ± 6.3 (17.9–44.1)	25.6 ± 4.4 (17.7–41.4)	24.8 (5.4) (16.8–43.6)	24.8 ± 5.5 (16.8–41.1)	0.026

### Statistical analysis

Normal distribution of the parameters was determined with the Kolmogorov–Smirnov test. Normally distributed parameters were compared with the student t-test or ANOVA test. Non-normally distributed parameters were compared using the Mann–Whitney U test or Kruskal–Wallis test. Interobserver reproducibility and intraobserver reliability were analyzed using intraclass correlation coefficient (ICC) [13]. All statistical tests were done using MedCalc (MedCalc Software Ltd, Ostend, Belgium Version 20.210).

## Results

### Relationship between topographical landmarks and anterior border of the gluteus maximus

Mean distance for parameter A was − 8.1 ± 1.0 cm, parameter B was 1.1 ± 1.0 cm, parameter C was 1.8 ± 1.2 cm, parameter D was 1.3 ± 0.9, and parameter E was 0.4 ± 0.9 cm (see Fig. 2).

### Landmark relationship categorized by age, gender, and femoral torsion

#### Age

When categorized by age < 40 and age ≥ 40 we observed the following: Parameters B, C, and D were significantly more posterior in the age ≥ 40 group compared to the age < 40 group. Parameters A and E were not significantly different between groups.

When further subdivided into groups by age (age < 20; age 20–40; age 40–60; age > 60), we observed the following: All parameters were significantly more posterior in the age > 60 group. A summary of findings is shown Tables 6 and 7.

**Table 6:** Results summary of subcategory: young vs. old

Parameter	Overall	Young	Old	p–value
A	− 8.1 ± 0.97 (− 11.1 to − 4.8)	− 8.1 ± 1.0 (10.5 to − 5.4)	− 8.2 ± 1.0 (− 11.1 to − 4.8)	0.3462
B	± 1.0 (− 2.0 to 4.9)	1.2 ± 1.0 (− 1.6 to 4.2)	1.0 ± 1.1 (− 2.0 to 4.9)	0.0071
C	1.8 ± 1.2 (− 3.4 to 6.0)	2.0 ± 1.0 (− 3.4 to 4.7)	1.6 ± 1.3 (− 2.0 to 6.0)	< 0.001
D	1.3 ± 0.9 (− 2.2 to 4.3)	1.5 ± 0.8 (− 1.6 to 4.0)	1.2 ± 1.0 (− 2.2 to 4.3)	< 0.001
E	0.4 ± 0.9 (− 2.9 to 3.2)	0.5 ± 0.8 (− 2.0 to 2.9)	0.3 ± 1.0 (− 2.9 to 3.2)	0.0775

**Table 7:** Results summary of subcategory: categorical age

Parameter	Overall	U20	20–40	40–60	> 60	p–value
A	− 8.1 ± 0.97 (− 11.1 to − 4.8)	− 8.1 ± 1.2 (− 10.5 to − 5.4)	− 8.1 ± 0.9 (− 10.4 to − 5.5)	− 8.1 ± 1.0 (− 10.7 to − 4.8)	− 8.5 ± 1.0 (− 11.1 to − 5.5)	0.00815
B	± 1.0 (− 2.0 to 4.9)	1.43 ± 0.8 (− 0.4 to 3.4)	1.2 ± 1.0 (− 1.6 to 4.2)	1.1 ± 1.0 (− 1.3 to 4.9)	0.7 ± 1.1 (− 2.0 to 4.1)	0.001
C	1.8 ± 1.2 (− 3.4 to 6.0)	2.0 ± 1.0 (− 0.4 to 4.3)	2.0 ± 1.0 (− 3.4 to 4.7)	1.8 ± 1.2 (− 2.0 to 6.0)	0.9 ± 1.2 (− 1.8 to 4.2)	< 0.001
D	1.3 ± 0.9 (− 2.2 to 4.3)	1.4 ± 0.9 (− 1.6 to 3.0)	1.5 ± 0.8 (− 1.5 to 4.0)	1.4 ± 1.0 (− 1.7 to 4.3)	0.6 ± 1.0 (− 2.2 to 3.0)	< 0.001
E	0.4 ± 0.9 (− 2.9 to 3.2)	0.5 ± 0.9 (− 1.8 to 2.1)	0.5 ± 0.8 (− 2.0 to 3.0)	0.4 ± 0.9 (− 2.9 to 3.2)	0.1 ± 1.1 (− 2.5 to 2.6)	0.006068

#### Gender

When comparing by gender we observed the following: Parameters A and E were significantly more anterior in females than in males. Parameters B, C, and D were not significantly different between females and males (Table 8).

**Table 8:** Results summary of subcategory: male vs. female

Parameter	Overall	Male	Female	p–value
A	− 8.1 ± 0.97 (− 11.1 to − 4.8)	− 9.0 ± 0.9 (− 11.1 to − 5.6)	− 7.7 ± 0.8 (− 10.0 to − 4.8)	< 0.001
B	± 1.0 (− 2.0 to 4.9)	1.1 ± 1.0 (− 2.0 to 4.9)	1.1 ± 1.0 (− 1.2 to 4.2)	0.9017
C	1.8 ± 1.2 (− 3.4 to 6.0)	1.7 ± 1.2 (− 3.4 to 6.0)	1.9 ± 1.1 (− 2.0 to 4.7)	0.0537
D	1.3 ± 0.9 (− 2.2 to 4.3)	1.2 ± 0.9 (− 2.2 to 4.3)	1.4 ± 0.8 (− 1.6 to 4.0)	0.0595
E	0.4 ± 0.9 (− 2.9 to 3.2)	0.3 ± 0.9 (− 2.5 to 3.2)	0.6 ± 0.8 (− 2.9 to 3.1)	< 0.001

#### Femoral torsion

Femoral torsion was subdivided into < 10°, 10°–25°, 25°–35°, and > 35°. A summary of findings is shown Table 9. We found that all parameters in group > 35° lay significantly more anterior compared to the other groups.

**Table 9:** Results summary of subcategory: categorical torsion

Parameter	U10	10–25	25–35	> 35	P–value
A	− 8.1 ± 0.9 (− 9.9 to − 6.4)	− 8.2 ± 1.0 (− 11.1 to − 5.4)	− 8.1 ± 1.0 (− 10.7 to − 4.8)	− 7.8 ± 0.9 (− 9.9 to − 5.6)	0.01
B	0.7 ± 0.9 (− 1.3 to 4.1)	1.1 ± 1.0 (− 2.0 to 3.8)	1.3 ± 1.0 (− 1.7 to 4.9)	1.4 ± 1.1 (− 1.9 to 4.2)	< 0.001
C	1.4 ± 1.0 (− 0.9 to 3.9)	1.8 ± 1.2 (− 3.4 to 6.0)	1.9 ± 1.0 (− 1.7 to 4.6)	2.0 ± 1.2 (− 1.3 to 4.5)	0.002
D	0.9 ± 1.0 (− 2.2 to 2.5)	1.2 ± 0.9 (− 1.9 to 4.3)	1.5 ± 0.8 (− 1.5 to 4.0)	1.4 ± 0.9 (− 1.2 to 3.4)	< 0.001
E	0.1 ± 1.0 (− 2.9 to 2.3)	0.4 ± 0.9 (− 2.8 to 3.2)	0.5 ± 0.7 (− 2.1 to 2.3)	0.8 ± 0.9 (− 2.0 to 3.1)	0.001

### Relationship between perforating vessels and the innominate tubercle

The 1st visible perforating vessel (PV) was a mean of 5.7 ± 3.1 cm proximal to the innominate tubercle. The 2nd PV was a mean of 8.2 ± 2.0 cm proximal. The 3rd PV was a mean of 9.1 ± 1.6 cm proximal. The 4th PV was 9.3 ± 1.5 cm proximal. 50% of the perforating vessels were found between 5 and 9 cm proximal to the innominate tubercle.

### Perforating vessel relationship categorized by age, gender, and femoral torsion

No statistically significant differences were noted in the location of any of the perforating vessels when stratifying patients by age, gender, or femoral torsion with the exception of the following: the 3rd perforating vessel was 1.2 cm more proximal in male patients (9.9 ± 1.5 cm) than in female patients (8.7 ± 1.5 cm; p = 0.0019), and the 2nd perforating vessel was located up to 1.4 cm more distal in very high torsion (7.2 ± 2.0 cm in torsion > 35°; 7.8 ± 2.4 cm in torsion 25°–35°; 8.5 ± 1.8 cm in torsion 10°–25°; 8.6 ± 2.0 cm in torsion < 10° vs; p = 0.0016).

## Discussion

**Fig. 5:**
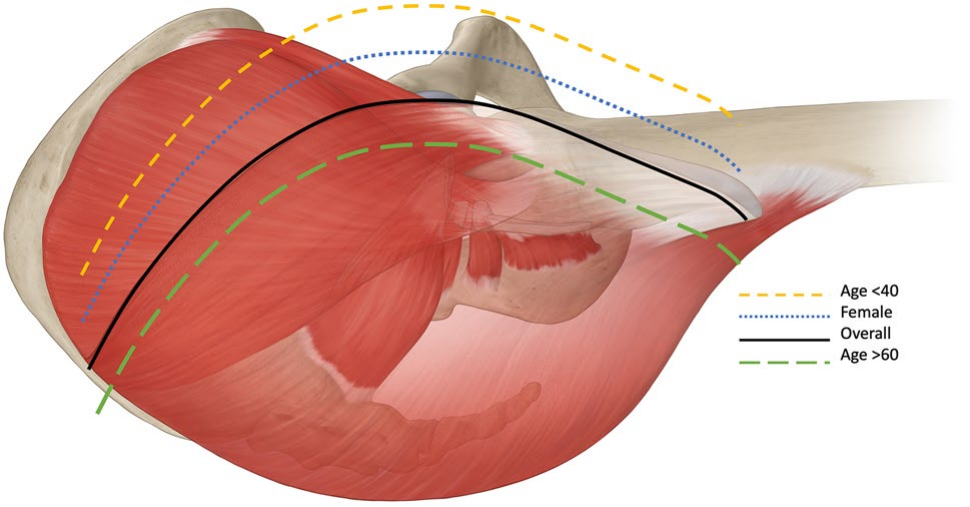
Visualization of Gibson interval in different sub-categories anterior to posterior (overall, < 40 years of age, female, > 60 years of age)

In every hip surgery we strive to minimize damage to muscle, soft tissue, and neurovascular structures. Accuracy when making an incision and precision when dissecting soft tissue is important. It can be especially valuable when anatomic studies provide clarity on factors that can influence how to perform a surgery with the best chance to minimize damage. This information not only provides predictability for the surgeon but can optimize post-operative results for the patient. In literature little is written about the position of the Gibson interval in different patient groups (Table 10). In this study we found that the Gibson interval is located more anteriorly in female patients and patients under 40 years of age. We also found that it is located more posteriorly in patients over 60 years of age. In addition, the interval moves anteriorly with increasing femoral torsion, most notably in patients with very high femoral torsion (> 35°) (Fig. 5).

**Table 10:** Literature table

First author	Year	Textbook/Paper	Specification
Gibson	1950	JBJS Br	First description of Gibson approach
Espinosa et al	2006	JBJS	Gluteus maximus in young athletic adults inserts into the iliotibial band, whereas it lies further posteriorly in elderly patients
Chomiak et al	2015	Hip International	With the posterior approach 71.4% of patients showed a lesion of the inferior gluteal nerve of the gluteus maximus muscle
Lawrenson et al	2019	Osteoarthritis and Cartilage	Muscle size is associated with bodyweight In bilateral osteoarthritis there is no difference in muscle size with controls. In unilateral osteoarthritis there is a smaller size of Glut. Minimus/Medius/Maximus in the symptomatic limb

Our study contains limitations. First, our measurements were done using CT imaging. This was not a cadaveric dissection with direct measurement to palpated landmarks. Also, all patients were imaged in the supine position with internal rotation of 15°, although the Gibson approach is performed with the patient in the lateral position. This could influence the distances, both relative and absolute, between the landmarks we evaluated and the anterior border of the gluteus maximus. We would expect that those influences would be uniform across different populations, however. Despite these limitations, we feel that the relationships we observed in this study reflect our clinical experience.

Femoral torsion seems to be a stronger factor in the location of the Gibson interval in younger patients than in older patients (age > 60). The gluteus maximus in young athletic adults inserts onto the iliotibial band, whereas it lies further posteriorly in elderly patients [6], which is in line with our findings. This may partly be due to atrophy of the gluteus maximus muscle with increasing age. Atrophy of the pelvis muscles is found in unilateral osteoarthritis, which would be more common in patients over 60 years of age [16, 18, 21]. While we did not assess the presence or absence of osteoarthritis in our patients, since all patients in our series underwent imaging for hip pain, we feel that some osteoarthritis is likely to be present in our cohort.

While this study shows that the interval moves more anteriorly with increasing femoral torsion, it is important to consider the potential confounding factors of femoral torsion and gender. Females, for instance, have increased femoral torsion compared to males [16]. In our study femoral torsion was 4.3 degrees higher in the female group (male 19.5° ± 9.2°, female 23.8° ± 11.2°, p < 0.001). Since the Gibson interval is located more anteriorly in both females and in patients with increasing femoral torsion it is not clear if one variable or the other carries more weight. Since parameter A measures the position relative to the pelvis and parameter E measures the position relative to the femur, we would expect a stronger anterior effect for parameter E than parameter A in patients with high femoral torsion. Indeed, this is what we observe. It appears that in females, while femoral torsion is a factor, it is also possible that the gynecoid pelvis affects the more proximal location of the Gibson interval.

Differences in pelvis morphology can influence the position of the Gibson interval since the gluteus maximus originates on the lateral iliac crest and inserts on the femur. These same differences also represent risk factors for acetabular retroversion (AR), which can be associated with retroversion of the entire hemipelvis, or with focal acetabular wall abnormalities, specifically posterior wall deficiency with anterior wall overcoverage [5]. While AR is present in 5 to 20% of the general population [11], it is more commonly seen in developmental dysplasia of the hip, Legg-Calvé-Perthes disease and Slipped Capital Femoral Epiphysis [7]. Parameter A, being located on the iliac wing, would function as an anatomic landmark that would vary with hemipelvis rotation. We did not specifically measure acetabular version in our study. While we feel that rotation of the hemipelvis would likely be a larger influence on the position of the Gibson interval, we cannot exclude isolated acetabular version as a potential confounding factor.

Our inclusion of the perforating vessels in this study provided additional information, and additional challenges. A cadaveric study described the presence of 4 to 6 perforating vessels present between the greater trochanter and the iliac crest [20]. In our cohort the median number of perforating vessels we observed per exam was one. 50% of the perforating vessels that we observed were situated between 5–9 cm proximal to the innominate tubercle. The position and number of perforating vessels may be influenced by patient factors such as obesity, dehydration, etc., but it is also notable that our 3D-MPR CT protocol was not optimized for vascular anatomy and did not go to the level of the iliac crest as Solomon described [20]. It is possible that by using ASIS as our upper limit we have not accounted for perforating vessels that lay proximal to ASIS, which can lead to an under representation. However, dissection proximally to ASIS during the Gibson approach is almost never necessary, limiting the influence of this on our results.

We found clear differences in the location of the Gibson interval in certain subgroups. These results were obtained based on CT scans in the supine position. To support these findings, we need intra-operative data, in the lateral position, to further evaluate the anatomical differences of the Gibson interval between subgroups as well as the position of the perforating vessels. Our goal was to provide surgeons with pre-operative information to guide incision placement and reduce the risk of excessive dissection or muscular trauma. In the future, we anticipate that preoperative imaging, coupled with technology such as augmented reality (AR) can be used to clearly define a patient-specific Gibson interval. While use of AR technology is rapidly evolving in orthopedic surgery, limitations in its current availability, as well as unresolved issues of safety and reliability, continue to make anatomy-based guidelines, like those presented in this study, useful for practicing surgeons [4, 17].

## Conclusion

There is a vast range of anatomical variation that can influence the position of the Gibson interval. We found the Gibson interval is located more anteriorly in female patients and patients under 40 years of age. We also found that it is located more posteriorly in patients over 60 years of age. In addition, the interval moves anteriorly with increasing femoral torsion, most notably in patients with very high femoral torsion (> 35°). Our results can be used to adjust our incision as well as where to expect the Gibson interval intra-operatively (as shown in Supplemental Digital Content 1).

## Supplementary Information

Below is the link to the electronic supplementary material.

Supplementary file1 (DOCX 22 KB)

## Data Availability

The authors confirm that the data supporting the findings of this study are available within its supplementary materials.
