# Supplementary material for: Can surface landmarks help us identify the gibson interval during surgical hip dislocation: a study of 617 hips
Source: Arch Orthop Trauma Surg. 2024 Dec 18;145(1):67. doi: 10.1007/s00402-024-05622-w (PMC11655569; doi:10.1007/s00402-024-05622-w)
Supplement: Supplementary file 1 — Supplementary file1 (DOCX 22 KB) [file 402_2024_5622_MOESM1_ESM.docx]

**Table 11.** Recommendations.

| Distance | Low torsion | | Normal torsion | | High torsion | |
| --- | --- | --- | --- | --- | --- | --- |
|  | Under 60 | Over 60 | Under 60 | Over 60 | Under 60 | Over 60 |
| Distance A  Median  50% (upper-lower quartile)  Standard deviation  2 x standard deviation  95% confidence interval | -8.02  -8.58 to -7.32  -8.89 to -7.15  -9.76 to -6.27  -8.20 to -7.76 | -8.52  -8.86 to -8.29  -9.27 to -8.06  -9.87 to -7.46  -9.09 to -8.24 | -8.14  -8.85 to -7.53  -9.18 to -7.19  -10.18 to -6.20  -8.30 to -8.07 | -8.40  -9.29 to -7.88  -9.65 to -7.48  -10.73 to -6.40  -8.90 to -8.23 | -7.91  -8.62 to -7.26  -8.98 to -6.98  -9.98 to -5.99  -8.12 to -7.84 | -8.13  -8.80 to -7.77  -9.04 to -7.41  -9.86 to -6.60  -8.55 to -7.91 |
| Distance B  Median  50% (upper-lower quartile)  Standard deviation  2 x standard deviation  95% confidence interval | 0.69  0.20 to 1.14  -0.10 to 1.54  -0.92 to 2.36  0.52 to 0.93 | 0.71  0.00 to 0.96  -0.74 to 2.09  -2.15 to 3.50  -0.34 to 1.69 | 1.12  0.48 to 1.73  0.18 to 2.13  -0.79 to 3.10  1.04 to 1.27 | 0.48  0.00 to 1.04  -0.59 to 1.50  -1.64 to 2.55  0.13 to 0.78 | 1.22  0.66 to 2.12  0.39 to 2.37  -0.60 to 3.35  1.24 to 1.51 | 1.23  0.50 to 1.87  0.00 to 2.15  -1.07 to 3.22  0.66 to 1.49 |
| Distance C  Median  50% (upper-lower quartile)  Standard deviation  2 x standard deviation  95% confidence interval | 1.49  0.83 to 2.25  0.56 to 2.40  -0.36 to 3.32  1.25 to 1.71 | 0.40  0.00 to 0.93  -0.62 to 1.97  -1.91 to 3.26  -0.25 to 1.60 | 1.99  1.25 to 2.70  0.78 to 3.09  -0.38 to 4.24  1.79 to 2.07 | 0.71  0.00 to 1.31  -0.52 to 1.89  -1.72 to 3.10  0.32 to 1.06 | 2.02  1.34 to 2.79  1.04 to 3.08  0.02 to 4.11  1.92 to 2.20 | 1.31  0.85 to 1.58  0.18 to 2.22  -0.84 to 3.25  0.81 to 1.60 |
| Distance D  Median  50% (upper-lower quartile)  Standard deviation  2 x standard deviation  95% confidence interval | 1.17  0.73 to 1.59  0.14 to 1.93  -0.76 to 2.83  0.81 to 1.26 | 0.53  -0.34 to 1.15  -1.04 to 1.56  -2.35 to 2.86  -0.67 to 1.19 | 1.40  0.84 to 1.90  0.61 to 2.23  -0.20 to 3.03  1.32 to 1.51 | 0.63  -0.32 to 0.91  -0.53 to 1.44  -1.52 to 2.43  0.15 to 0.76 | 1.42  1.08 to 2.12  0.79 to 2.33  0.02 to 3.10  1.46 to 1.67 | 1.11  0.63 to 1.70  0.08 to 2.00  -0.89 to 2.96  0.66 to 1.41 |
| Distance E  Median  50% (upper-lower quartile)  Standard deviation  2 x standard deviation  95% confidence interval | 0.48  -0.53 to 0.76  -0.82 to 1.09  -1.78 to 2.04  -0.10 to 0.37 | -0.43  -1.54 to 0.56  -1.88 to 1.048  -3.35 to 2.51  -1.47 to 0.63 | 0.51  0.00 to 0.85  -0.44 to 1.28  -1.30 to 2.14  0.31 to 0.52 | 0.00  -0.67 to -0.55  -0.90 to 0.9878  -1.85 to 1.93  -0.25 to 0.33 | 0.64  0.22 to 1.11  -0.08 to 1.38  -0.81 to 2.10  0.54 to 0.75 | 0.45  -0.15 to 1.00  -0.84 to 1.29  -1.91 to 2.36  -0.19 to 0.64 |
| Different subgroups and their absolute values in cm. Positive values are located anterior of a reference landmark, negative values are located posterior of a reference landmark. | | | | | | |
